# Navigating the Landscape of MANF Research: A Scientometric Journey with CiteSpace Analysis

**DOI:** 10.1007/s10571-023-01412-x

**Published:** 2023-09-26

**Authors:** Caixia Zhang, Mi Zhang, Xueqin Cao, Bo Jiao, Wencui Zhang, Shangchen Yu, Xianwei Zhang

**Affiliations:** 1 Department of Anesthesiology, Tongji Hospital, Tongji Medical College, Huazhong University of Science and Technology, 1095 Jie Fang Avenue, Wuhan, 430030 Hubei People’s Republic of China; 2 Department of Anesthesiology, Zhongnan Hospital, Wuhan University, Wuhan, Hubei People’s Republic of China

**Keywords:** MANF, Mesencephalic astrocyte-derived neurotrophic factor, ER, CiteSpace, Bibliometric

## Abstract

This study employs bibliometric analysis through CiteSpace to comprehensively evaluate the status and trends of MANF (mesencephalic astrocyte-derived neurotrophic factor) research spanning 25 years (1997–2022). It aims to fill the gap in objective and comprehensive reviews of MANF research. MANF-related studies were extracted from the Web of Science database. MANF publications were quantitatively and qualitatively analyzed for various factors by CiteSpace, including publication volume, journals, countries/regions, institutions, and authors. Keywords and references were visually analyzed to unveil research evolution and hotspot. Analysis of 353 MANF-related articles revealed escalating annual publications, indicating growing recognition of MANF's importance. High-impact journals such as the *International Journal of Molecular Sciences* and *Journal of Biological Chemistry* underscored MANF’s interdisciplinary significance. Collaborative networks highlighted China and the USA’s pivotal roles, while influential figures and partnerships drove understanding of MANF's mechanisms. Co-word analysis of MANF-related keywords exposed key evolutionary hotspots, encompassing neurotrophic effects, cytoprotective roles, MANF-related diseases, and the CDNF/MANF family. This progression from basic understanding to clinical potential showcased MANF’s versatility from cellular protection to therapy. Bibliometric analysis reveals MANF’s diverse research trends and pathways, from basics to clinical applications, driving medical progress. This comprehensive assessment enriches understanding and empowers researchers for dynamic evolution, advancing innovation, and benefiting patients.

**Graphical Abstract:**

Bibliometric analysis of MANF research. The graphical abstract depicts the bibliometric analysis of MANF research, highlighting its aims, methods, and key results.

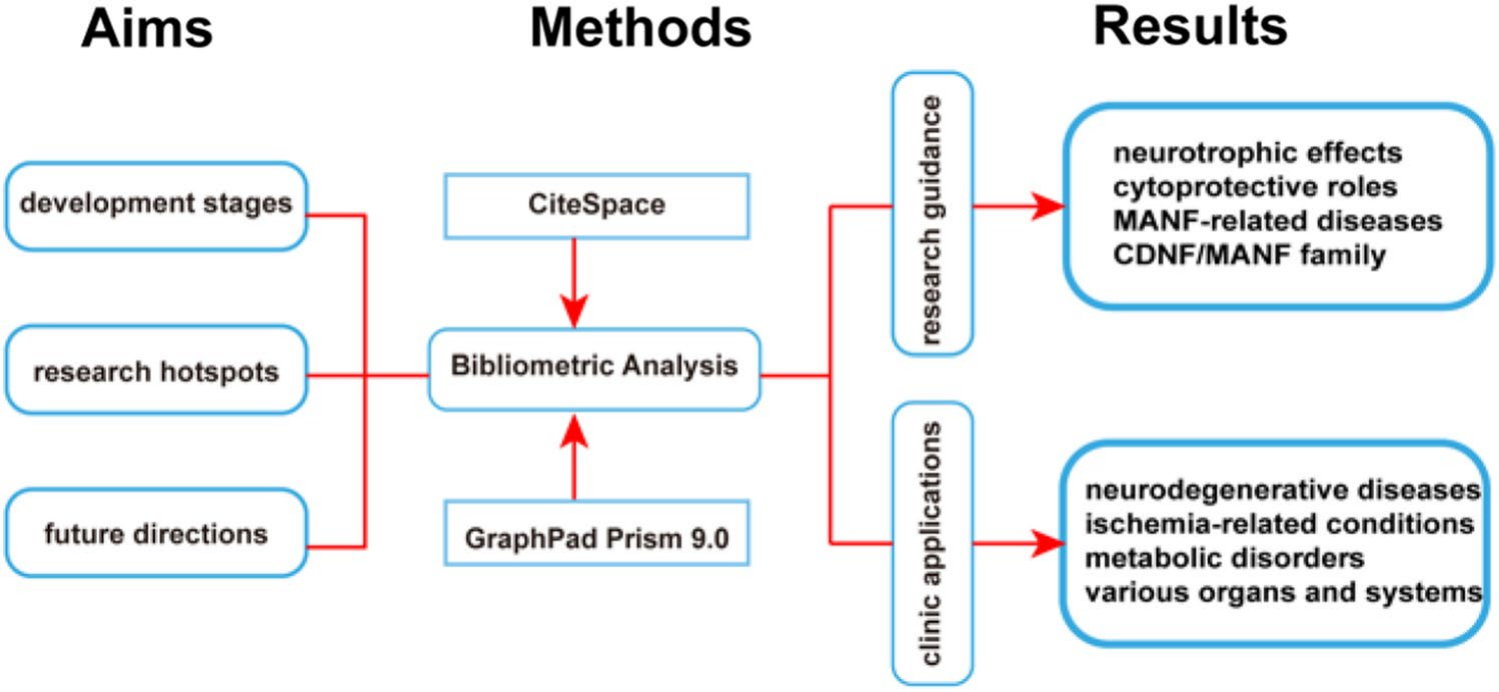

## Introduction

Mesencephalic astrocyte-derived neurotrophic factor (MANF) also known as arginine-rich mutated in early tumors (ARMET) (Petrova et al. 2003; Sanderson et al. 2009; Shridhar et al. 1996) or arginine-rich protein (ARP) (Evron et al. 1997) was initially discovered in 2003, is an emerging neurotrophic factor (NTFs) in vertebrates that has gained significant attention in medical research (Petrova et al. 2003). MANF exhibits distinct characteristics compared to other NTFs, displaying minimal sequence similarity and representing an evolutionarily ancient protein (Petrova et al. 2003; Palgi et al. 2009b; Bai et al. 2018). It has garnered interest due to its wide expression in both invertebrate and vertebrate species (Wang et al. 2021b, 2014; Lindholm et al. 2008; Chen et al. 2012; Palgi et al. 2009a), as well as its cytoprotective effects on neuronal and non-neuronal cell survival and development (Lindholm et al. 2008; Wang et al. 2021b; Kim et al. 2017). MANF plays a crucial role in various physiological processes, particularly during early developmental stages (Wen et al. 2022). Its expression is prominently observed in the central nervous system (Wang et al. 2021b; Kim et al. 2017), where it serves as a protective factor against neuronal degeneration and apoptosis (Lindholm and Saarma 2022; Wang et al. 2021b). Additionally, MANF influences neurite outgrowth and extension (Tseng et al. 2017; Wen et al. 2020), modulates neuron differentiation (Paolino et al. 2018; da Silva and Dotti 2002), and preserves cell migration (Wang et al. 2021b; Kim et al. 2017). Beyond the nervous system, MANF is also expressed in active secretory and metabolic tissues, such as the pancreas, liver, hypothalamus, and pituitary gland (Lindholm et al. 2008; Liu et al. 2015), where it contributes to maintaining metabolic homeostasis and mitigating inflammation (Imran et al. 2017; Sousa-Victor et al. 2019; Liu et al. 2020; Tang et al. 2022).

Emerging evidence suggests that MANF exhibits transiently increased levels and participates in the pathogenesis of diverse diseases, providing a protective effect (da Silva and Dotti 2002; Xu et al. 2019; Liu et al. 2021; Wang et al. 2021b; Yu et al. 2021; Axelsen and Woldbye 2018). These diseases include neurodegenerative disorders, such as Parkinson’s and Alzheimer’s diseases (da Silva and Dotti 2002; Xu et al. 2019; Liu et al. 2021; Wang et al. 2021b; Yu et al. 2021; Axelsen and Woldbye 2018), spinocerebellar ataxia (SCA) (Danilova and Lindahl 2018; Guo et al. 2018; Yang et al. 2014a), Central Nervous System (CNS) injuries and stroke (Zhao et al. 2013; Yang et al. 2022; Caglayan et al. 2022; Belayev et al. 2020; Lindholm et al. 2008), autoimmune (Fonseca et al. 2011; Morito and Nagata 2012), cancer (Peled et al. 2021; Alam et al. 2021), metabolic diseases (Tang et al. 2022; Wang et al. 2020b; Yu et al. 2021; Lindholm et al. 2006; Apostolou et al. 2008; Fonseca et al. 2011; Mätlik et al. 2018), and glomerular and tubular nephropathy (Inagi et al. 2014; Morito and Nagata 2012). While the experimental verification of MANF has primarily been limited to certain disease models (Wang et al. 2021b; Yu et al. 2021; Axelsen and Woldbye 2018; Eesmaa et al. 2022), the promising evidence thus far suggests its potential as a therapeutic target for these conditions (Axelsen and Woldbye 2018; Kim et al. 2017; Montaser et al. 2021b; Wang et al. 2021b; Yu et al. 2021).

Amid the growing volume of MANF-related research, understanding the field’s orientation remains challenging. Reanalyzing relevant MANF publications using CiteSpace, a web-based Java application, proves vital (Chen et al. 2010). CiteSpace facilitates analysis and visualization of Web of Science data (Chen et al. 2010), generating collaborative maps (Chen 2006; Chen et al. 2010). This study employs CiteSpace to conduct a bibliometric analysis of MANF research from 1997 to 2022, evaluating aspects, like publications, journals, countries/regions, institutions, authors, keywords, and references. The goal is to provide a comprehensive overview of MANF’s evolution, research trends, and potential future directions. Ultimately, this analysis enhances our understanding of MANF and guides future research.

## Data and Methods

### Data Collection

The data for the bibliometric analysis were collected from Clarivate Analytics’ Web of Science Core Collection using the following index terms: ‟mesencephalic astrocyte-derived neurotrophic factor” OR ‟MANF” OR ‟arginine-rich mutated in early tumors (ARMET)” OR ‟arginine-rich protein (ARP).” The data were collected from January 1, 1997 to November 1, 2022, resulting in a total of 353 studies, including 33 reviews and 320 research articles. The downloaded data included authors, titles, keywords, abstracts, and citations, which were then inputted into CiteSpace for further analysis. The search records were downloaded on November 1, 2022.

### Inclusion Criteria

1. Original articles and reviews on MANF.
2. Articles published between January 1, 1997 and November 1, 2022.
3. Articles available in the Web of Science database.
4. Articles published in English.

### Exclusion Criteria

1. Unofficial publication of articles.
2. Articles collected through manual methods or phone communication.
3. Conference proceedings, abstracts, and corrigendum documents.
4. Duplicate publications.
5. Articles not relevant to the topic.

### Quality Assessment

Only English articles that met the inclusion criteria and did not meet the exclusion criteria were included in the analysis.

### Analysis Method

**Fig. 1:**
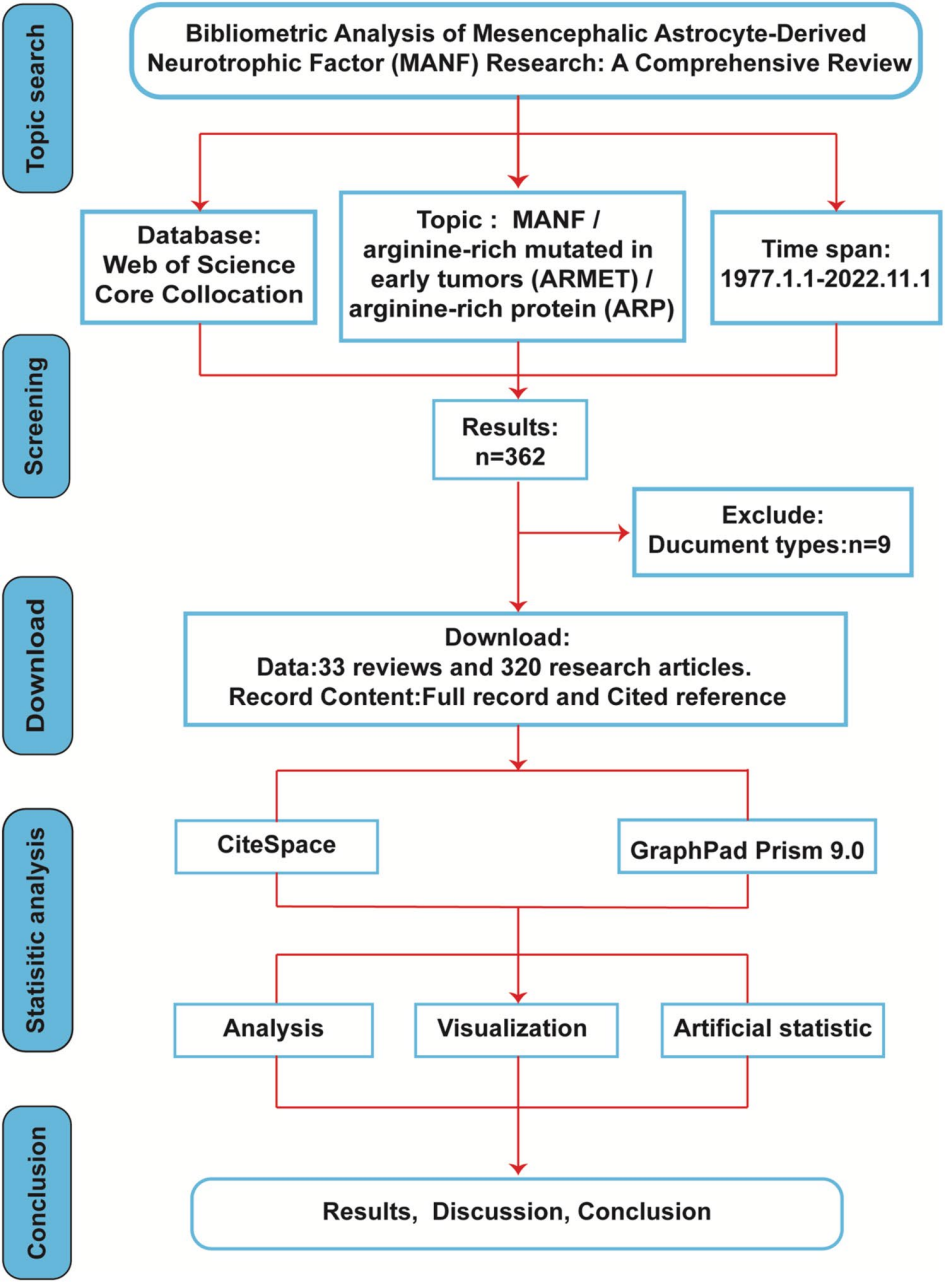
Flowchart of scientometric analysis in MANF

The workflow of scientometric research (Fig. 1) outlines the process. Using CiteSpace 6.1.R3W software, the MANF literature analysis covered 1997 to 2022 in 1-year segments. Multiple sources like titles, abstracts, keywords, authors, institutions, and countries were considered, with a threshold of top = 50. Node size denoted citations, color indicated co-occurrence or co-citation time, and cable thickness represented relationship strength. Centrality identified key points. The critical path method visualized elements, such as keywords, authors, and publications. Co-occurrence maps unveiled trends over time, while the time zone view captured evolving relationships. GraphPad Prism 9.0 analyzed data, countries, institutions, authors, and Strategic Coordinate Diagrams.

## Results

### Quantitative Analysis of Basic Information

#### The Trend of Annual Publications

**Fig. 2:**
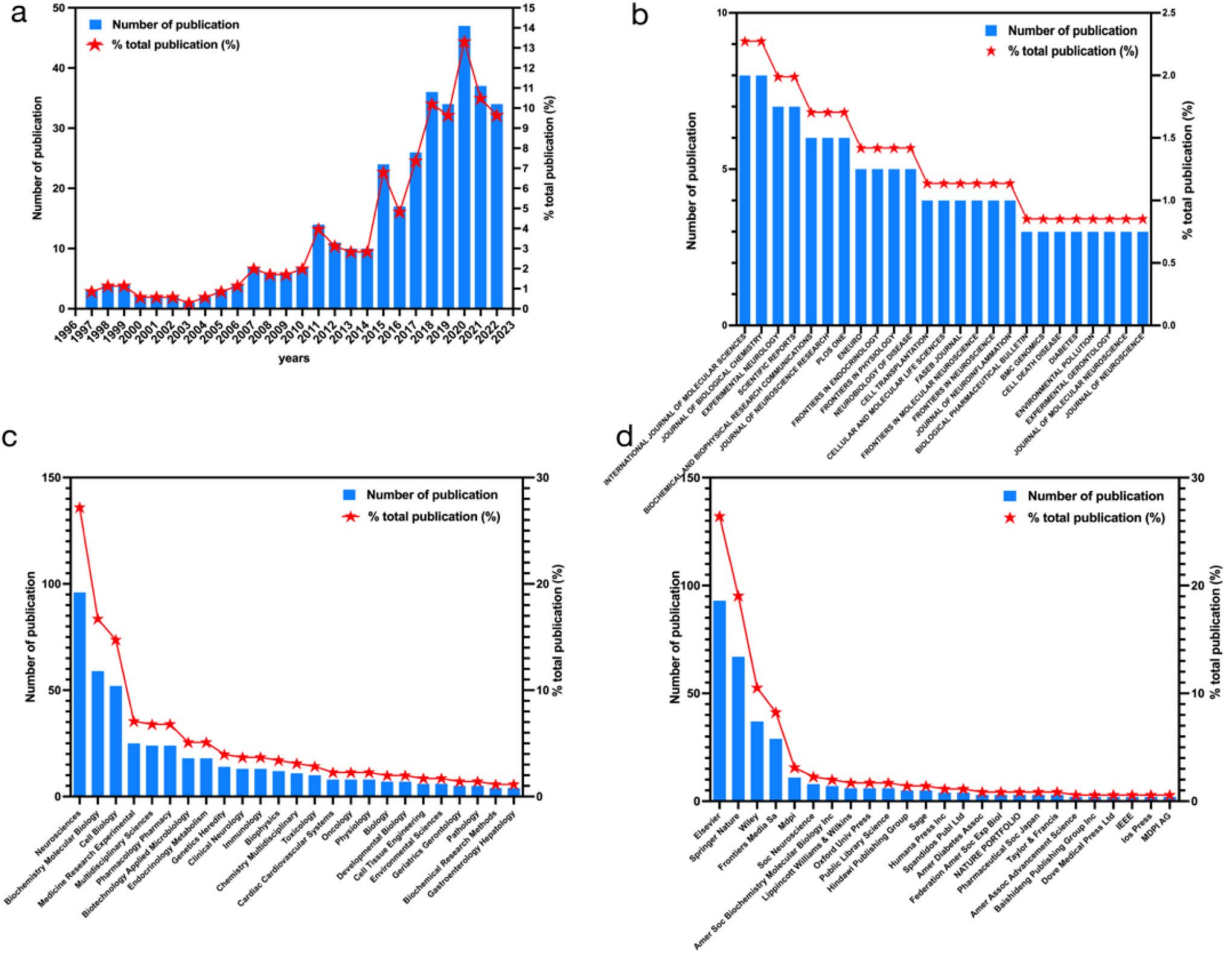
**a** Annual publications in the field of MANF. **b** The top 25 journals in the field of MANF. **c** The top 25 Web of Science subject classifications in the field of MANF. **d** The top 25 publication units in the field of MANF

Figure 2a displays the annual publication volume trend for papers related to MANF from January 1, 1997 to November 1, 2022. The data reveals a steady increase in the number of publications on MANF over the years. Since 2011, there have been over 10 articles per year, with a peak of 47 articles in 2020. While the growth in publication volume was relatively slow between 1997 and 2010, there has been exponential growth since 2013. This surge can be attributed to advancements in basic medical technology and the persistent exploration of MANF’s neuroprotective applications in cell cultures and animal models. As of November 1, 2022, a total of 34 articles have been published, and it is expected that more will follow. The upward trajectory of annual publications underscores the active and promising nature of MANF research.

### Published Journal

We identified the top 25 journals based on publication volume in Fig. 2b, out of a total of 202 journals. The *International Journal of Molecular Sciences* and the *Journal of Biological Chemistry* were tied for first place with 8 publications each. They were followed by *Experimental Neurology and Scientific Reports* with 7 publications each and *Biochemical and Biophysical Research Communications* and *Journal of Neuroscience Research* both had 6 publications. Figure 2c presents the top 25 Web of Science subject classifications in the field of MANF, *Neurosciences* ranked first with 96 publications, followed by *Biochemistry and Molecular Biology* with 59 publications. The 25th category was *Biochemical Research Methods* and *Gastroenterology Hepatology* with 4 publications. In terms of publishing units, as shown in Fig. 2d, Elsevier ranked first with 93 publications, followed by Springer Nature with 67, Wiley with 37, *Frontiers Media Sa* with 29, MDPI with 11, and *Soc Neuroscience* with 8 publications. Based on the above analysis, it is evident that MANF research primarily focuses on *Neurology and Biochemistry*, and most publications are associated with Elsevier and Springer Nature. While the publication peak in this field may not be as high as in other areas, the value and significance of the MANF field are increasingly recognized by scientific researchers.

### The Network of Cooperative Relationship

#### The Cooperation Network of Countries and Institutions

Our statistical analysis focused on MANF-related papers from various countries and regions, aiming to identify influential institutions and their collaborative networks. Between 1997 and 2022, around 44 countries and regions contributed to MANF-related publications. Notably, Table 1 highlights the top 10 countries and regions in terms of publication volume, China leads with 138 articles, followed by the USA with 98. China’s Cluster ID of 12.5 in 2006 suggests concentrated publishing activity, while the USA’s Cluster ID of 19.5 hints at early involvement in MANF research. Finland achieved a Cluster ID of 10.5 in 2007 and Japan reached 13.5 in 1998, indicating their contributions and clustering patterns.

**Table 1:** The top 10 countries and regions in the field of MANF

Number	Countries	Counts	Years	Centrality	Burst	Sigma	Cluster ID
1	China	138	2006	0.27	0	1	12.5
2	USA	98	1997	0.82	5.24	22.94	19.5
3	Finland	73	2007	0.31	0	1	10.5
4	Japan	25	1998	0.03	5.39	1.17	13.5
5	England	21	2002	0.26	0	1	12.5
6	Canada	17	1998	0.08	0	1	19.5
7	Estonia	15	2007	0	0	1	9.5
8	Sweden	14	1999	0.04	0	1	16.5
9	Germany	13	1998	0.13	0	1	20.5
10	France	6	1997	0.17	0	1	21.5

**Fig. 3:**
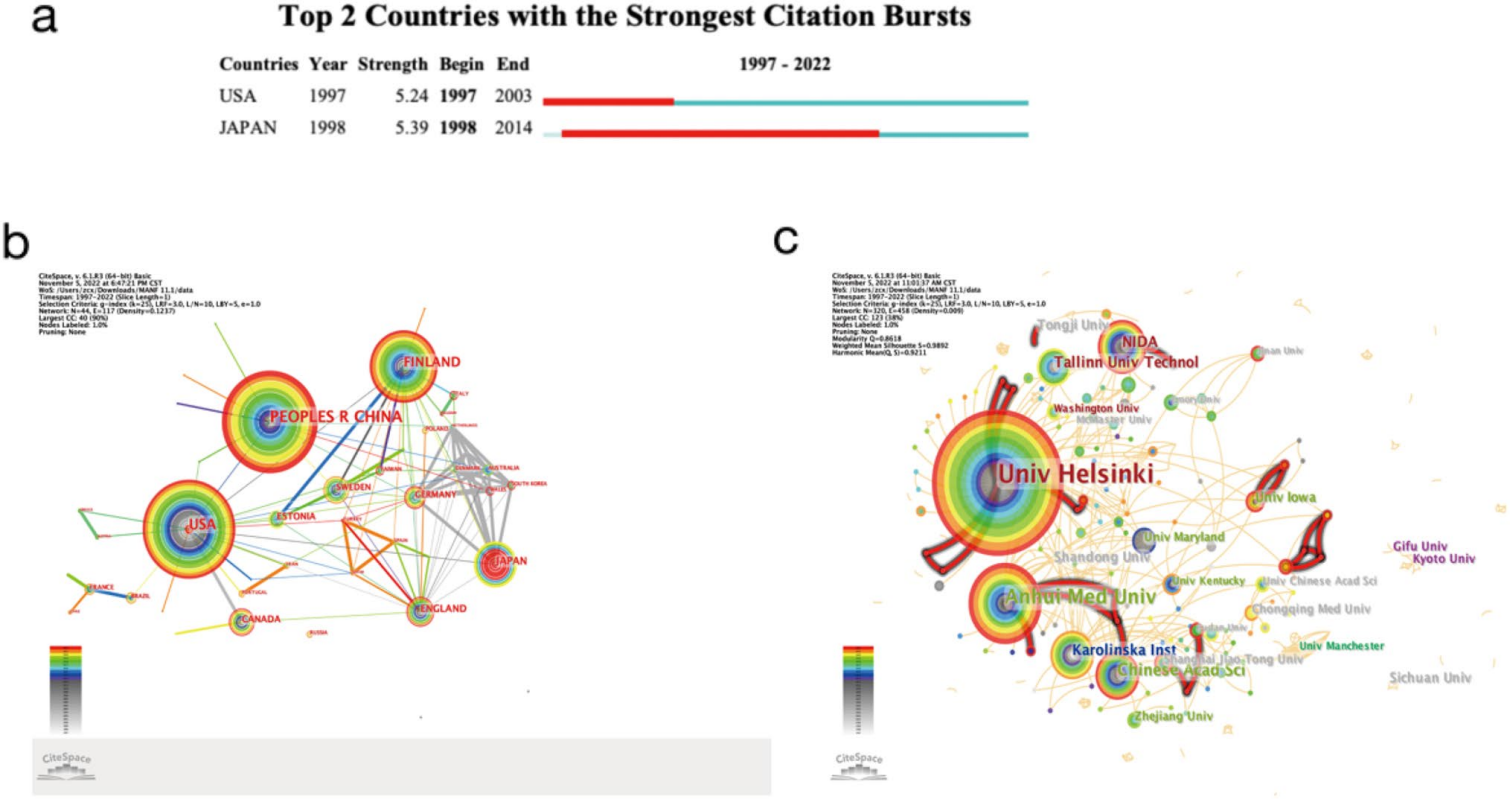
**a** The top 2 countries and regions with the strongest citation bursts in the field of MANF. **b** The cooperative relationship between countries and regions in the field of MANF. **c** The cooperative relationship between institutions in the field of MANF

Figure 2a highlights countries with the strongest citation bursts. The USA exhibits a burst strength of 5.24 from 1997 to 2003 and Japan records a burst strength of 5.39 from 1998 to 2014, reflecting their historical strengths in MANF research. Figure 3b portrays cooperative relationships among countries. China, the USA, Finland, and Japan, characterized by higher publication volumes, have larger nodes. Notably, robust collaborations are evident between Japan and Finland, denoted by thicker connecting lines. Centrality analysis ranks the USA highest, trailed by Finland and China, underlining their influential roles and strong field connections.

Regarding institutional cooperation, we examined 321 publishing institutions (Table 2; Fig. 3c). The top 10 institutions include five from China and two from the USA, reinforcing their dominance. The University of Helsinki (Finland) leads with 73 publications, while others also have significant contributions. Notably, Anhui Medical University (China) and the Chinese Academy of Sciences (China) are prominent publishers. Collaboration analysis unveils close ties among key contributors. The University of Helsinki and the Chinese Academy of Sciences hold central roles (centrality values of 0.2 and 0.13), emphasizing their influence in MANF research. The study highlights China and the USA’s leading roles in both publication volume and institutional impact, offering insights into the global MANF research landscape.

**Table 2:** The top 10 publishing institutions in the field of MANF

Number	Counts	Degree	Centrality	Institutions	Country	Year
1	73	48	0.2	University of Helsinki	Finland	2007
2	33	17	0.04	Anhui Medical University	China	2008
3	13	18	0.13	Chinese Academy of Sciences	China	2006
4	11	9	0.03	National Institute on Drug Abuse	USA	2007
5	11	6	0	Tallinn University of Technology	Estonia	2007
6	10	20	0.04	Karolinska Institute	Sweden	1999
7	9	2	0	Tongji University	China	2016
8	8	7	0.03	The University of Iowa	USA	2011
9	8	5	0	Shandong University	China	2013
10	8	3	0	Sichuan University	China	2017

#### The Network of Author Cooperation

**Fig. 4:**
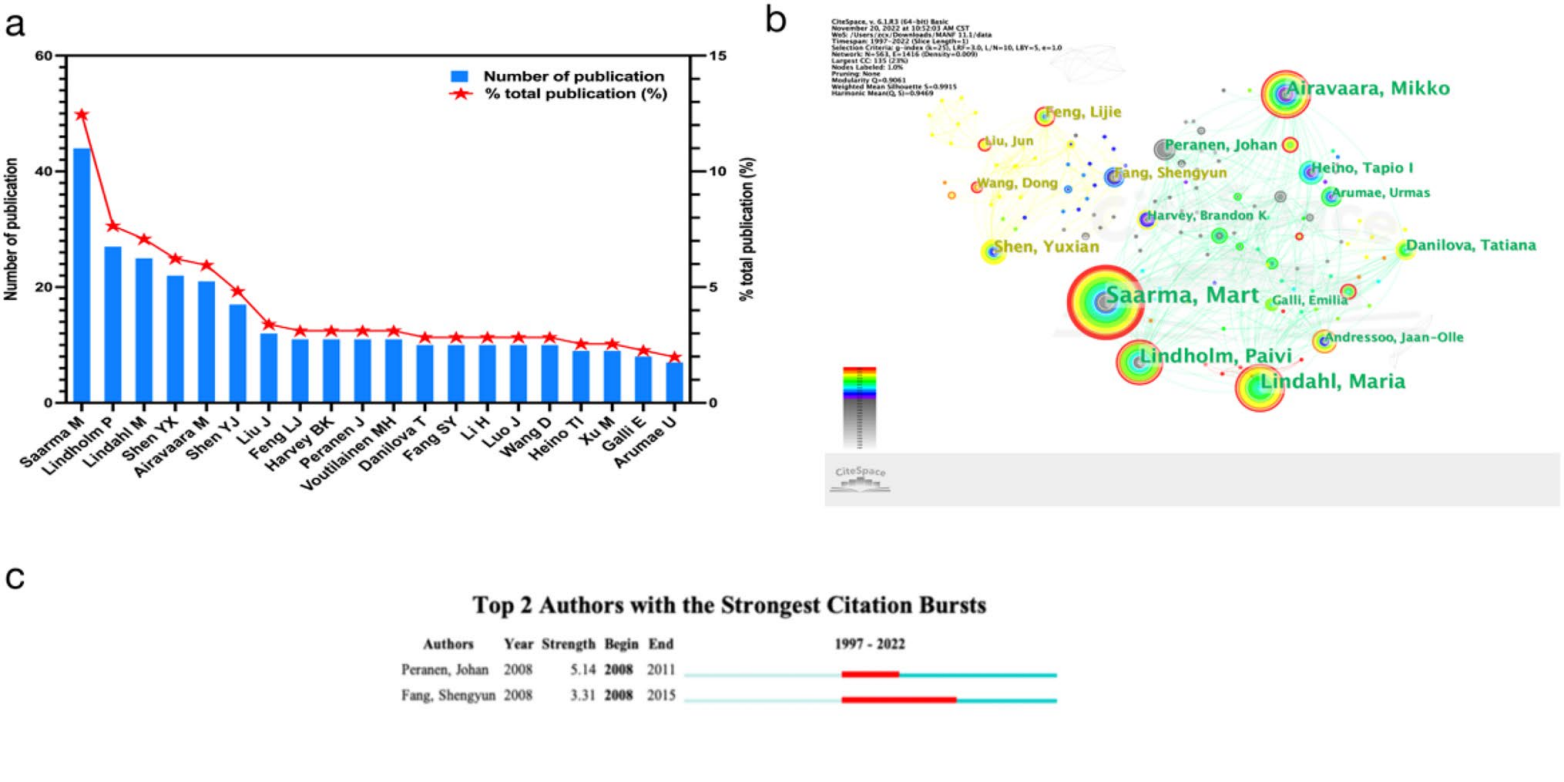
**a** The top 20 authors in the field of MANF. **b** The cooperative relationship between authors in the field of MANF. **c** The top 2 authors with the strongest citation bursts in the field of MANF

Using CiteSpace, we analyzed the top 20 authors (Fig. 4a; Table 3) out of 563 authors involved in MANF research. Professor Saarma Mart from the University of Helsinki ranked first with 44 articles, followed by Lindholm from Northwestern University with 27 articles, and then Thrie Lindahl Maria from the University of Linköping with 25 articles. Examining their cooperation, we observed a decentralized author network, with closer relationships among a few scholars and larger groups remaining more distant (Fig. 4b). These cooperative relationships were primarily based on institutional or academic affiliations.

**Table 3:** The top 10 authors in the field of MANF

Number	Authors	Counts	Institutions	Countries
1	Saarma Mart	44	University of Helsinki	Finland
2	Lindholm Paivi	27	Northwestern University	USA
3	Lindahl Maria	25	University of Linköping	Sweden
4	Shen YuXian	22	Sichuan University	China
5	Airavaara Mikko	21	University of Helsinki	Finland
6	Shen YuJun	17	Tianjin Medical University	China
7	Liu Jun	12	Anhui Medical University	China
8	Feng LiJie	11	Anhui Medical University	China
9	Harvey Brandon K	11	National Institute on Drug Abuse	USA
10	Peranen Johan	11	University of Helsinki	Finland

The top three authors maintained a long-term partnership focusing on the function and mechanism of neurotrophic factors MANF and CDNF in various diseases (Kovaleva and Saarma 2021; Eesmaa et al. 2021; Kovaleva et al. 2023; Voutilainen et al. 2015; Cordero-Llana et al. 2015; Lindholm and Saarma 2010; Lindahl et al. 2017, 2014). Their work unveiled MANF’s ER-based cytoprotective function, interacting with the UPR sensor IRE1α or aiding GRP78 (Eesmaa et al. 2021; Kovaleva et al. 2023) to regulate UPR and calcium homeostasis (Pakarinen et al. 2022; Lindholm and Saarma 2022). They explored MANF’s role in ER stress, apoptosis, and neuronal degeneration (Lindholm et al. 2008; Kovaleva and Saarma 2021), as well as its role in beta cell survival and regeneration (Lindahl et al. 2014). Absence of MANF relates to ER stress-triggered outer hair cell death and deafness (Herranen et al. 2020). These insights underscore MANF’s value in neuronal and non-neuronal cell survival, hinting at its therapeutic potential for ER stress-linked disorders (Fonseca et al. 2011; Morito and Nagata 2012; Herranen et al. 2020; Lindahl et al. 2017, 2014).

Another notable collaboration is observed between Fang Shengyun, Shen Yuxian, and Wangdong, scrutinizing MANF’s impact on macrophages and visceral function (Shen et al. 2022). Notably, the analysis pinpoints authors with strong citation bursts (Fig. 4c), like Peranen Johan’s burst strength of 5.24 from 2008 to 2011, and Fang Shengyun’s burst strength of 5.39 from 2008 to 2015, suggesting their forthcoming influence in MANF research. The collaborative endeavors of these authors have significantly advanced MANF comprehension, its mechanisms, therapeutic applications, and its relevance across various disorders. Sustained collaboration and knowledge exchange among these authors will propel the field further.

In summary, our analysis of basic information in MANF research has shown a steady rise in annual publications, particularly since 2013, driven by advances in medical technology. Key journals and subject categories underscore the multidisciplinary nature of this field. Collaborative networks highlight the leading roles of China and the USA, with institutions like the University of Helsinki and authors such as Professor Saarma Mart contributing significantly. This information equips researchers with insights to align their work strategically, emphasizing collaboration and multidisciplinary approaches. It offers a compass for navigating the evolving MANF landscape, guiding new directions and innovative applications.

## Hotspot Evolution Analysis

### Keywords

**Fig. 5:**
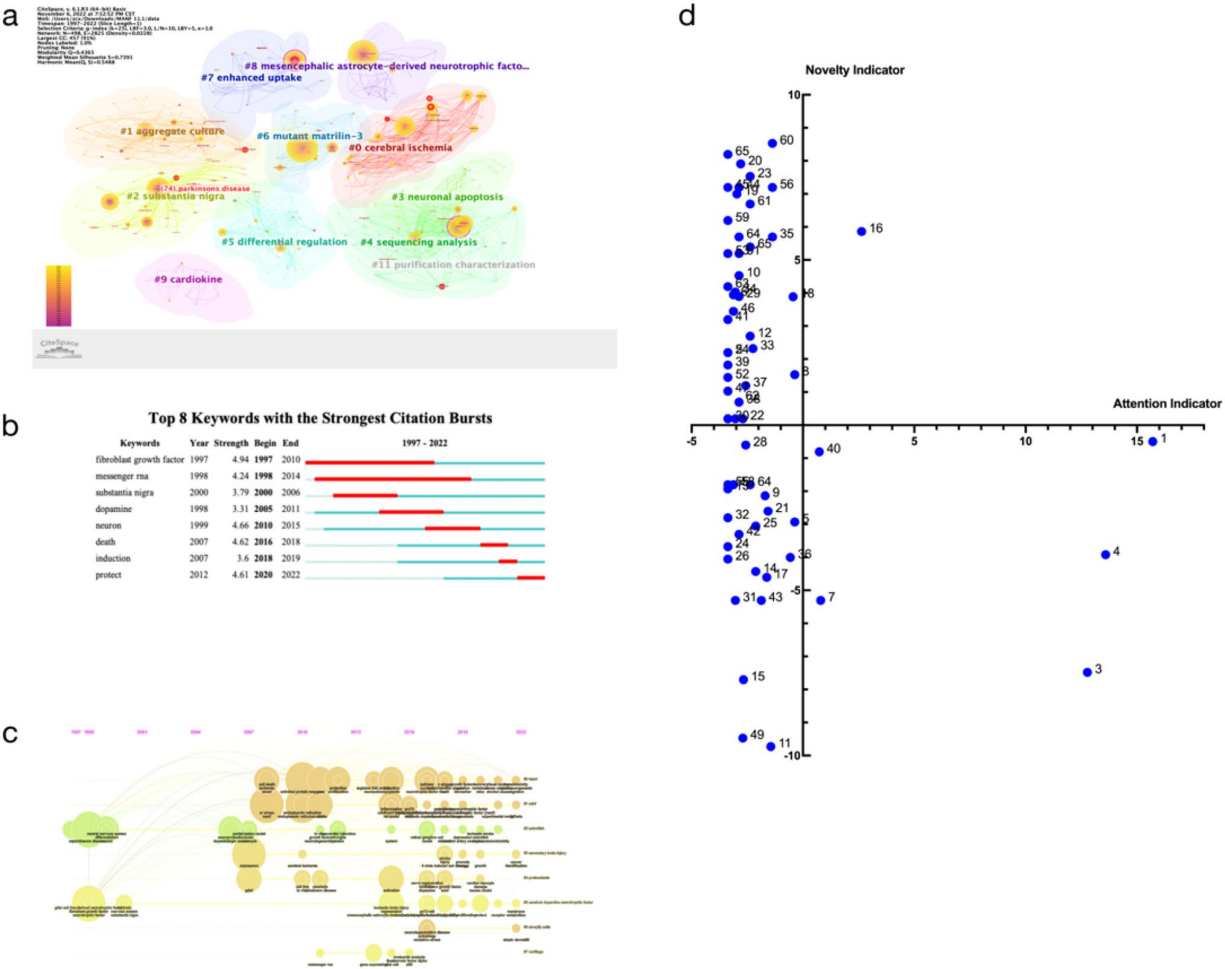
**a** The cooperative relationship between keywords in the field of MANF. **b** The top 8 keywords with the strongest citation bursts in the field of MANF. **c** Keywords timeline view in the field of MANF. **d** Strategic coordinate diagram in the field of MANF

Leveraging CiteSpace, we dissected keyword distribution to unveil evolving trends in MANF research. The top 10 keywords were identified (Table 4), with MANF taking the lead at 148 occurrences and centrality of 0.08 since 2001. Prominent keywords encompassed endoplasmic reticulum, neurotrophic factor, unfolded protein response, Parkinson’s disease, dopamine, gene expression, rat, and cell. Clustering categorized MANF articles, unearthing 11 sub-clusters of co-occurring keywords, outlining distinct research directions (Fig. 5a). The knowledge map depicted the centrality–frequency relationship among keywords. Furthermore, keyword bursts were spotted, unveiling cutting-edge topics cited frequently over specific intervals (Fig. 5b). Bursting keywords included fibroblast growth factor (4.94 strength, 1997–2010), messenger RNA (4.24 strength, 1998–2014), substantia nigra (3.79 strength, 2000–2006), dopamine (3.31 strength, 2005–2011), neuro (4.66 strength, 2010–2015), death (4.62 strength, 2016–2018), induction (3.6 strength, 2018–2019), and protect (4.61 strength, 2020–2022). These bursts mirror the dynamic essence of MANF research, signifying the ascendancy of specific themes as their significance gains traction.

**Table 4:** The top 10 keywords in the field of MANF

Number	Keywords	Counts	Degree	Centrality	Year
1	MANF	148	64	0.08	2001
2	Endoplasmic reticulum	129	76	0.07	2007
3	Neurotrophic factor	105	80	0.13	1997
4	UPR	76	68	0.07	2010
5	Parkinson’s disease	74	83	0.08	1998
6	Dopamine	62	122	0.26	1998
7	Gene	61	106	0.2	1997
8	Expression	59	80	0.11	2007
9	Rat	51	67	0.05	1997
10	Cell	47	107	0.15	1997

By combining the keyword timeline view and co-citation patterns (Fig. 5c), we observed a shift in MANF research focus over time. Initially, research primarily centered on the cytoprotective effects of MANF, particularly in dopamine neurons. However, recent years have seen a shift toward investigating MANF's clinical benefits and potential therapeutic applications across various diseases. This expansion of focus signifies MANF’s growing relevance as a potential treatment strategy beyond neuroprotection.

The analysis of keyword co-occurrence and bursts provides valuable insights into research hotspots and the evolving landscape of MANF research. It highlights the broadening scope of research from cytoprotection to potential clinical applications in different diseases.

### Strategic Coordinate Diagram in the Field of MANF

We constructed a strategic coordinate diagram based on re-analyzed keyword data from CiteSpace to predict future research hotspots and trends in the field of MANF. The diagram consisted of four quadrants representing different research clusters (Fig. 5d; Table 5). In the first quadrant, we identified core research clusters with high novelty and attention indicators. The CDNF/MANF family (16#) emerged as a focal point, indicating its ongoing significance in research. The second quadrant encompassed potential research clusters with higher novelty but lower attention indicators, suggesting future hotspots and areas of development. These clusters covered a wide range of topics, including brain barrier dysfunction (58#), metabolism disease (20#), cell injury (23#), oxidase deficiency (19#), pathogenesis (62#), convection enhanced delivery (63#), and various disease-specific clusters. Clusters in the third quadrant represented marginal research areas with lower novelty and attention indicators. Shifting focus from these clusters to more promising areas might be beneficial. Examples included differentiation (11#), receptor (49#), re-innervation (26#), hippocampal neuron (24#), Acute coronary syndrome (56#), and cell therapy (28#). The fourth quadrant consisted of basic research clusters with higher attention but lower novelty indicators. These clusters focused on foundational studies in areas, such as endoplasmic reticulum (1#), neurotrophic factor (3#), cytoprotective (4#), endothelial growth factor (7#), and CDNF (40#).

**Table 5:** Name of clusters in Fig. 5c

Number	Name of cluster	Attention indicator	Novelty indicator
1	Endoplasmic reticulum	15.71	− 0.50
2	Cerebral energy failure	− 3.37	2.19
3	Neurotrophic factor	12.77	− 7.48
4	Cytoprotective	13.59	− 3.92
5	Drug seeking	− 0.37	− 2.93
6	Constructive heuristics	− 3.12	3.94
7	Endothelial growth factor	0.80	− 5.31
8	Oxidative stress	− 0.37	1.53
9	Akt pathway	− 1.70	− 2.14
10	Huntington’s disease	− 2.87	4.53
11	Differentiation	− 1.45	− 9.73
12	Acute kidney injury	− 2.37	2.69
13	Axon formation	− 3.37	− 1.93
14	Mechanical injury	− 2.12	− 4.43
15	Neurodegenerative disease	− 2.67	− 7.71
16	CDNF/MANF family	2.63	5.86
17	Multiple system atrophy	− 1.63	− 4.61
18	Inflammation	− 0.45	3.89
19	Oxidase deficiency	− 2.97	6.99
20	Metabolism disease	− 2.80	7.91
21	6 hydroxydopamine	− 1.57	− 2.61
22	Adeno-associated virus	− 2.70	0.19
23	Cell injury	− 2.37	7.53
24	Hippocampal neuron	− 3.37	− 3.68
25	Prevents apoptosis	− 2.12	− 3.06
26	Re-innervation	− 3.37	− 4.06
28	Cell therapy	− 2.57	− 0.61
29	Nerve regeneration	− 2.87	3.89
30	Antidepressant	− 3.37	0.19
31	Reduces apoptosis	− 3.04	− 5.30
32	Network state adaptive	− 3.37	− 2.80
33	Blood–brain barrier	− 2.24	2.31
34	Depression	− 3.03	4.02
35	Intracellular trafficking	− 1.36	5.69
36	Acyclic form	− 0.57	− 4.01
37	Calcium homeostasis	− 2.57	1.19
38	Necrosis factor alpha	− 2.87	0.69
39	Asymmetric supercapacitor	− 3.37	1.82
40	CDNF	0.73	− 0.81
41	Cardiovascular risk	− 3.36	3.19
42	Cell survival	− 2.86	− 3.30
43	Biosynthesis	− 1.86	− 5.30
44	Bemisia tabaci	− 2.86	7.19
45	Adsorption	− 3.36	7.19
46	Ciliary neurotrophic factor	− 3.12	3.44
47	Extracellular polysaccharide	− 3.37	1.03
49	Receptor	− 2.70	− 9.47
51	Cognitive deficit	− 3.37	1.44
52	Brain damage	− 3.36	5.19
53	6-ohda rat model	− 3.36	2.19
54	Cholesterol	− 3.36	− 1.80
55	Inhibition	− 1.36	7.19
56	Acute coronary syndrome	− 3.12	− 1.81
58	Brain barrier dysfunction	− 1.37	8.53
59	Caenorhabditis elegan	− 2.36	6.69
60	CN	− 3.04	0.19
61	Histone deacetylase	− 3.36	4.19
62	Pathogenesis	− 2.87	5.69
63	Convection enhanced delivery	− 2.37	5.39
64	Developmental neurotoxicity	− 2.36	− 1.80
65	Adipose tissue	− 3.36	8.19

The strategic coordinate diagram provides researchers with a roadmap for future exploration. Core research clusters, such as the CDNF/MANF family, remain central and warrant continued investigation. Additionally, potential research clusters with high novelty indicators, like brain barrier dysfunction, metabolism diseases, and cell injury, offer exciting avenues for further inquiry. These clusters represent emerging hotspots where researchers can make novel contributions. Additionally, research has highlighted the involvement of MANF in various diseases and pathogenic processes across multiple tissues and systems. Future research hotspots are expected to explore the therapeutic potential of MANF in different diseases (Yang and Gao 2020; Liu et al. 2021; Kovaleva and Saarma 2021), including neurodegenerative diseases (Liu et al. 2021; Kovaleva and Saarma 2021), brain barrier dysfunction (Gao et al. 2020), metabolism diseases (Danilova et al. 2019; Cordero-Llana et al. 2015), acute kidney injury (Yang and Gao 2020; Liu et al. 2021; Kovaleva and Saarma 2021), cognitive deficits (Liu et al. 2022; Zhang et al. 2023), depression (Liu et al. 2022; Zhang et al. 2023), inflammation (Sun et al. 2022; Liu et al. 2022; Zhang et al. 2023, 2022), and cardiovascular risk (Zhang et al. 2022).

Conversely, marginal research areas with lower novelty and attention indicators may indicate areas where shifting focus could be advantageous. Investigating extracellular and intracellular mechanisms related to MANF, such as cell injury, oxidase deficiency, convection-enhanced delivery, calcium homeostasis, extracellular polysaccharides, and intracellular trafficking, will also be important. This approach ensures that research efforts are directed toward promising directions with potential real-world applications.

Ultimately, researchers can leverage these insights to guide their studies and contribute to the advancement of MANF research. By delving deeper into the mechanisms of the CDNF/MANF family, exploring therapeutic applications, and investigating emerging hotspots, researchers have the opportunity to uncover new knowledge and potentially revolutionize disease treatment strategies. The evolving landscape of MANF research provides a dynamic platform for scientific innovation and discovery.

## Analysis of Reference

**Fig. 6:**
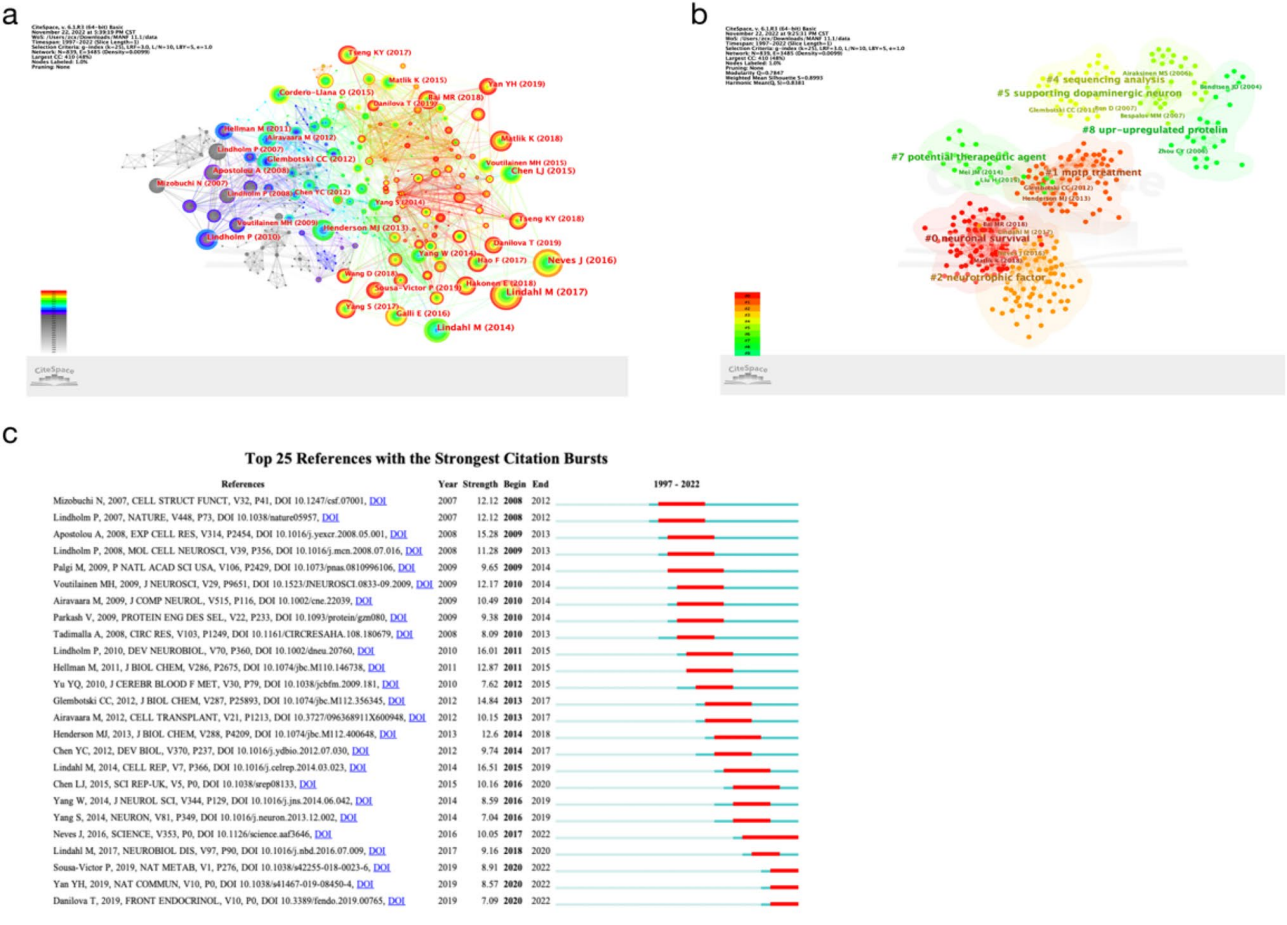
**a** The cooperative relationship between references in the field of MANF. **b** The cooperative relationship between reference clusters in the field of MANF. **c** The top 25 references with the strongest citation bursts in the field of MANF

The analysis of references in the field of MANF provides valuable insights into citation patterns and influential works (Table 6). One notable reference that has garnered significant interest is Richman et al. (2018), with the highest frequency of 66 citations. This reference holds importance and impact, as indicated by its half-life of 1.5 and centrality of 0.02. Figure 6a illustrates the cooperative relationships among MANF references over time, with the increasing number and size of nodes representing growing influence and collaboration among researchers. The color of the nodes represents the referenced years, while the intricate connection lines depict the complexity of the relationships between references. Arranging chronologically from left to right shows an increasing number and size of nodes over time, along with a growing quantity and complexity of connection lines. The intricate connection lines signify the complexity of these relationships.

**Table 6:** The top 10 references in the field of MANF

Number	Freq.	Burst	Centrality	Sigma	Author	Year	Source	Half-life
1	66	0	0.02	1	Richman et al. (2018)	2018	*Front Neurosci-Switz*	1.5
2	56	4.96	0.02	1.1	Bai et al. (2018)	2018	*Nat Commun*	1.5
3	51	4.45	0.04	1.18	Nadella et al. (2014)	2014	*J Neuroinflamm*	2.5
4	39	6.08	0.03	1.22	Lindström et al. (2013)	2013	*PLoS One*	2.5
5	33	0	0.01	1	Garea-Rodríguez et al. (2016)	2016	*PLoS One*	2.5
6	32	0	0	1	Hartman et al. (2019)	2019	*Eur J Cell Biol*	1.5
6	32	0	0.01	1	Mätlik et al. (2017)	2017	*eNeuro*	1.5
8	31	3.49	0.01	1.04	Voutilainen et al. (2017)	2017	*eNeuro*	1.5
8	31	6.9	0.02	1.17	Mätlik et al. (2015)	2015	*Cell Death Dis*	2.5
10	30	0	0.02	1	Cunha et al. (2017)	2017	*J Biol Chem*	2.5
10	30	5.51	0.03	1.17	Cordero-Llana et al. (2015)	2015	*Mol Ther*	2.5

A chronological analysis of the references highlights several significant works in the field. Notable references include Voutilainen et al. (2009), Lindholm and Saarma (2010), Glembotskl et al. (2012), Airavaara et al. (2012), Henderson, Lindahl et al. (2014), Chen et al. (2015), Cordero-Liana et al. (2015), Neves, Lindahl et al. (2017), Tsang et al. (2017), Matlik et al. (2018), and Yan et al. (2020). These references contribute to the evolving knowledge and understanding of MANF. Figure 6b presents a clustering network of references, grouping them based on subject areas or focal points. Each cluster represents a specific topic or theme, with core references marked for their high centrality and frequency. The sub-clusters cover various aspects of MANF research, such as neuronal survival (0#), MPTP treatment (1#), neurotrophic factors (2#), and more.

Figure 6c highlights references with high burst values, indicating emerging trends in MANF research from 2008 to 2022. Lindahl et al. (2014) stands out with a burst value of 16.51, confirming MANF’s potential to enhance and restore β-cell proliferation (Lindahl et al. 2014). Another significant burst value (16.01) is associated with Lindholm and Saarma (2010), suggesting MANF’s potential as a therapeutic approach for neurodegenerative diseases by promoting the survival, development, maintenance, and differentiation of neurons (Lindholm and Saarma 2010). Overall, the analysis of references provides valuable insights into the influential works, emerging trends, and research directions in the field of MANF. Researchers can refer to these references to stay updated on the latest developments and contribute to the advancement of MANF research.

## Discussion

In this thorough analysis of 353 MANF-related articles spanning 1977 to 2022, quantitative and visual techniques were employed to unveil the field’s diverse dimensions. Trends in annual publications reflected a consistent rise, underlining growing recognition of MANF’s significance. Notably, prestigious journals such as the *International Journal of Molecular Sciences* and *Journal of Biological Chemistry* featured prominently, highlighting MANF’s interdisciplinary nature. Collaborative networks revealed China and the USA’s pivotal roles, while influential figures like Professor Saarma Mart and impactful partnerships drove understanding of MANF’s mechanisms (Lindahl et al. 2017; Pakarinen et al. 2022; Lindholm and Saarma 2022; Danilova et al. 2019). Authors with citation bursts, like Peranen Johan and Fang Shengyun, indicated their potential influence (Palgi et al. 2009a; Xu et al. 2019). This comprehensive analysis offers a broad perspective on MANF research, aiding future endeavors, collaborations, and guiding stakeholders navigating this evolving landscape.

The co-word analysis of MANF-related keywords revealed key hotspots driving the field’s evolution. These include the endoplasmic reticulum’s role, MANF as a neurotrophic factor, its cytoprotective effects, focus on MANF-related diseases and their mechanisms, and exploration of the CDNF/MANF protein family. This analysis highlights a progression from basic understanding to clinical applications, underscoring MANF’s potential in diverse contexts, from cellular protection to therapeutic interventions. Foundational studies served as beacons, MANF’s initial recognition stemmed from its exceptional capacity to enhance the survival of dopaminergic neurons in vitro.

This intricate network of pathways conferred upon MANF an array of valuable properties, ranging from promoting cell survival, thwarting apoptosis (Zhang et al. 2017), and bolstering antioxidant defenses to regulating autophagy and facilitating neurite outgrowth (Liu et al. 2022; Sun et al. 2017; Voutilainen et al. 2015). Notably, the subsequent sections offer an in-depth exploration of MANF’s multifaceted roles and the underlying mechanisms driving its protective effects across a spectrum of diseases (Liu et al. 2022).

MANF offers diverse neuroprotection in neurodegenerative diseases and ischemia. In Parkinson’s models, MANF manages cellular stress via endoplasmic reticulum regulation (Apostolou et al. 2008; Yu et al. 2021) and activates antioxidant pathways through PI3K/Akt/GSK3β and AMPK/mTOR pathway to enhances mitochondrial function (Apostolou et al. 2008; Oh-Hashi et al. 2012; Yang et al. 2020). Notably, elevated blood levels of MANF in Parkinson’s disease patients suggest its potential as a diagnostic biomarker (Fu et al. 2021). Similarly, in ischemia, MANF plays a role in curbing neuronal apoptosis by orchestrating UPR-related genes (such as GRP78, phosphorylated IRE1, and XBP1s) (Yang et al. 2014b) and activating the Akt/MDM2/P53 pathway (Airavaara et al. 2009; Zhao et al. 2013). Moreover, MANF promotes neurite outgrowth through the Akt/mTOR and Erk/mTOR pathways (Airavaara et al. 2009; Zhao et al. 2013). Its influence extends to the blood–brain barrier, where it preserves tight junctions and dampens inflammation via TLR4/MyD88/NF-κB pathways (Han et al. 2022). Additionally, MANF contributes to post-ischemic recovery through pro-angiogenic effects and increased cerebral blood flow, supported by its interaction with vascular endothelial growth factor (VEGF) (Gao et al. 2020). The multifaceted neuroprotection offered by MANF underscores its potential as a promising avenue for further research in neurodegenerative diseases and ischemia-related conditions.

Beyond its neuroprotective roles, MANF plays a pivotal role in maintaining metabolic equilibrium. Its connection to age in type 1 diabetes (T1D) patients highlights its significance in preserving β-cell function (Weir and Bonner-Weir 2013). MANF counters ER stress-induced impairment and fights inflammation-triggered apoptosis by repressing NF-κB (Hakonen et al. 2018). Moreover, MANF finely tunes hypothalamic insulin signaling via PIP4k2b, influencing food intake and body weight (Hakonen et al. 2018; Montaser et al. 2021a). This intervention results in decreased obesity and inhibition of fatty acid biosynthesis and cholesterol production, contributing to a healthier metabolic profile. MANF’s diverse functions position it as a promising candidate for therapeutic interventions in metabolic disorders.

Additionally, MANF’s versatility extends to various organs and systems. In heart disease, it addresses cardiac ischemia-induced ER stress (Glembotski 2011), reduces tissue damage in myocardial infarction (Glembotski et al. 2012), and mitigates atrial apoptosis in chronic atrial fibrillation (Wang et al. 2020a). MANF also inhibits bacterial myocarditis and modulates M1 macrophage differentiation (Wang et al. 2021a). Within the liver, its overexpression maintains metabolic balance (Sousa-Victor et al. 2019), counters fatty acid-induced steatosis (He et al. 2020) and protects against hepatic ischemia/reperfusion injury by inhibiting pro-apoptotic pathways (Yang et al. 2021). It suppresses alcohol-induced liver injury and exhibits anticancer properties in hepatocellular carcinoma via NF-κB/Snail pathway inhibition (Liu et al. 2020). In kidney disease, MANF’s role in ER stress-related cell signaling gains prominence (Kim et al. 2016), particularly in nephrotic syndrome and glomerular/tubular disorders (Kim et al. 2016; Tousson-Abouelazm et al. 2020). Recent findings highlight its immune regulation during acute kidney injury through mono-macrophage-derived MANF (Tousson-Abouelazm et al. 2020). In the spleen, MANF uniquely impacts immune cells, notably macrophages, shaping splenic immune dynamics (Liu et al. 2015). It engages with cochlear hair cells, vital for auditory fidelity (Herranen et al. 2020). In retinal pathologies, MANF promotes optic neurite growth, enhances retinal ganglion cell survival/functionality, and guards against oxidative stress (Wang et al. 2020b). It counters hyperglycemia-induced ER stress and apoptosis through Akt signaling, offering extensive research opportunities (Wang et al. 2020b).

Regarding the CDNF/MANF family’s pivotal role highlighted by the strategic coordinate diagram analysis, their discovery in the early 2000s has not led to a complete grasp of their fundamental biology and cytoprotective mechanisms (Lindahl et al. 2017; Pakarinen et al. 2022). Notably, both CDNF and MANF display neuroprotective effects that extend beyond Parkinson’s disease, embracing conditions like cerebral ischemia and spinocerebellar ataxia (Lindholm and Saarma 2010). Leveraging knockout models of these proteins across diverse organisms holds the promise of unveiling their multifaceted functions and therapeutic potential across a wide spectrum of neurodegenerative disorders (Pakarinen et al. 2022; Kordower and Bjorklund 2013). In conclusion, the comprehensive analysis of keyword distribution and the strategic coordinate diagram provides invaluable insights into evolving research trends, emerging areas, and potential pathways, thus significantly enriching the dynamic panorama of MANF research.

The analysis of MANF-related references uncovers important trends and impactful works. Richman (2018) reference stands out for its high citations, indicating its importance. Noteworthy works like Voutilainen et al. (2009) and Lindholm and Saarma (2010) emerge in the chronological view, enriching MANF understanding. Burst trends, exemplified by Lindahl et al. (2014) and Lindholm and Saarma (2010), underscore MANF’s potential in β-cell growth and neurodegenerative disease treatment. This analysis guides researchers in tracking trends and advancing MANF research.

In conclusion, this comprehensive analysis provides valuable insights into MANF research trends, emerging areas, and potential directions. The dynamic nature of MANF’s roles, from fundamental mechanisms to clinical applications, promises exciting possibilities for medical advancements. Researchers are empowered to contribute significantly to the ever-evolving landscape of MANF, ultimately benefiting patients and medical progress.

## Limitation

This study’s quantitative approach using the Web of Science database has limitations. It might miss relevant research from other sources. The analysis timeframe and language focus might exclude recent developments and non-English research, narrowing the scope of findings.

## Data Availability

The datasets presented in this study can be found online (Clarivate Analytics’ Web of Science Core Collection).
